# Supplementary material for: In Vitro and Ex Vivo Evaluation of Mangifera indica L. Extract-Loaded Green Nanoparticles in Topical Emulsion against Oxidative Stress and Aging
Source: Biomedicines. 2022 Sep 13;10(9):2266. doi: 10.3390/biomedicines10092266 (PMC9496353; doi:10.3390/biomedicines10092266)
Supplement: Supplementary file 1 [file biomedicines-10-02266-s001.zip › biomedicines-1847827-supplementary.pdf]

Supporting Information

80(0.5) 20/m-125(0.5) 3/m-260 (2)Inj255 aux270 **, 15-Jun-2021 + 14:55:55**

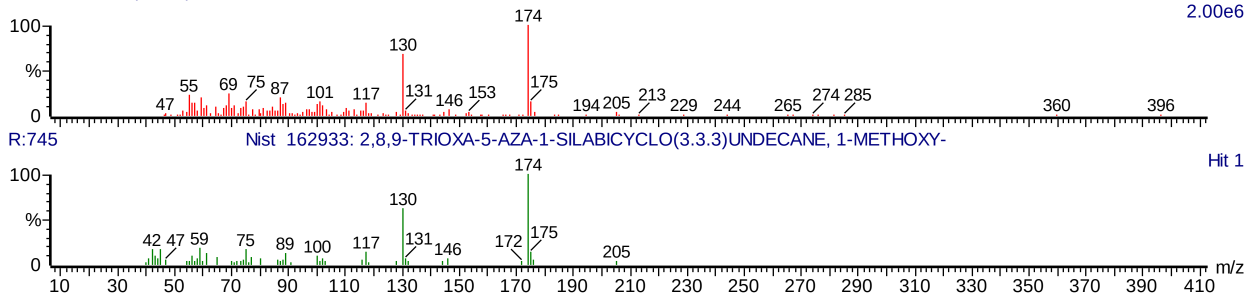

Hit	Rev	For	Compound Name	M.W.	Formula	CAS	Library
1	745	543	2,8,9-TRIOXA-5-AZA-1-SILABICYCLO(3.3.3)U	205	C7H15O4NSi	4025-80-3	Nist

80(0.5) 20/m-125(0.5) 3/m-260 (2)Inj255 aux270 **, 15-Jun-2021 + 14:55:55**

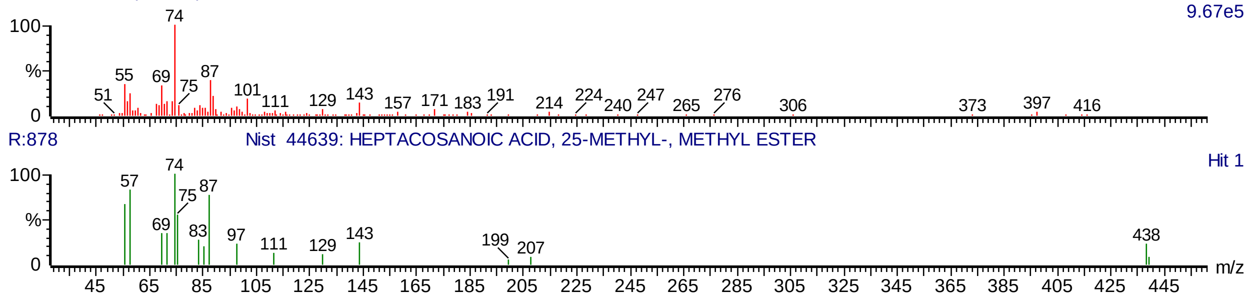

Hit	Rev	For	Compound Name	M.W.	Formula	CAS	Library
1	878	561	HEPTACOSANOIC ACID, 25-METHYL-, METHYL E	438	C29H58O2	900112-14-5	Nist

80(0.5) 20/m-125(0.5) 3/m-260 (2)Inj255 aux270 , **15-Jun-2021 + 14:55:55**

zahirm800 1151 (12.257)

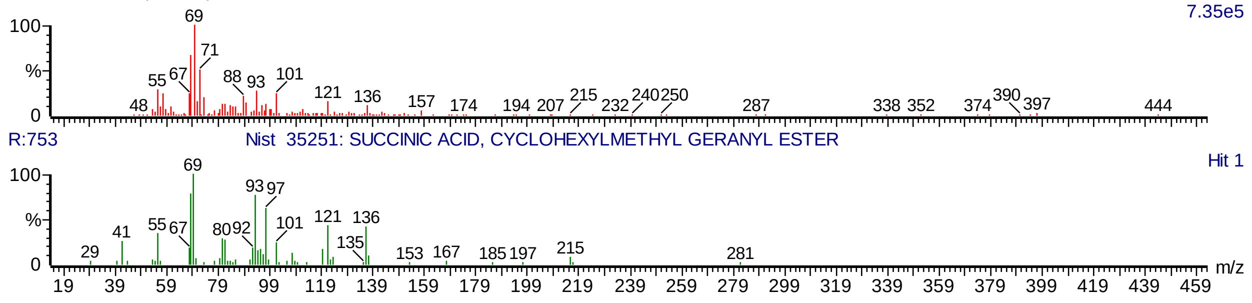

Hit	Rev	For	Compound Name	M.W.	Formula	CAS	Library
1	753<br>Nist	517	SUCCINIC ACID, CYCLOHEXYLMETHYL GERANYL	350	$C_{21}H_{34}O_4$	900391-21-5	

80(0.5) 20/m-125(0.5) 3/m-260 (2)Inj255 aux270 , **15-Jun-2021 + 14:55:55**

zahirm800 1265 (12.828)

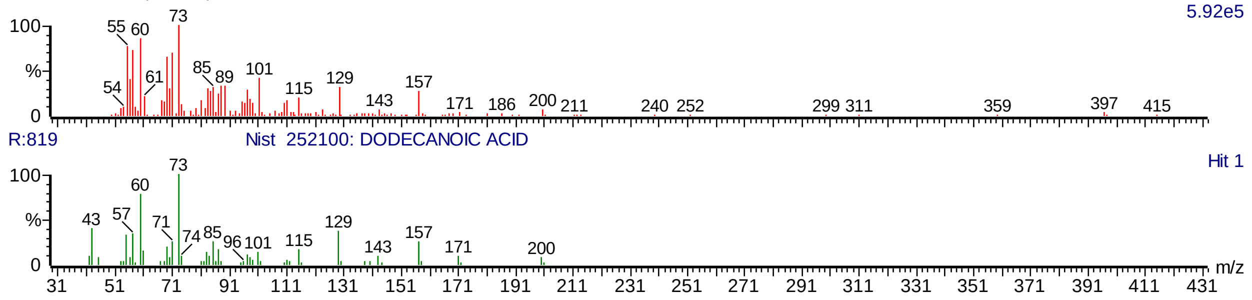

Hit	Rev	For	Compound Name	M.W.	Formula	CAS	Library
1	819	704	DODECANOIC ACID	200	$C_{12}H_{24}O_2$	143-07-7	Nist

80(0.5) 20/m-125(0.5) 3/m-260 (2)Inj255 aux270 , **15-Jun-2021 + 14:55:55**

zahirm800 1474 (13.873)

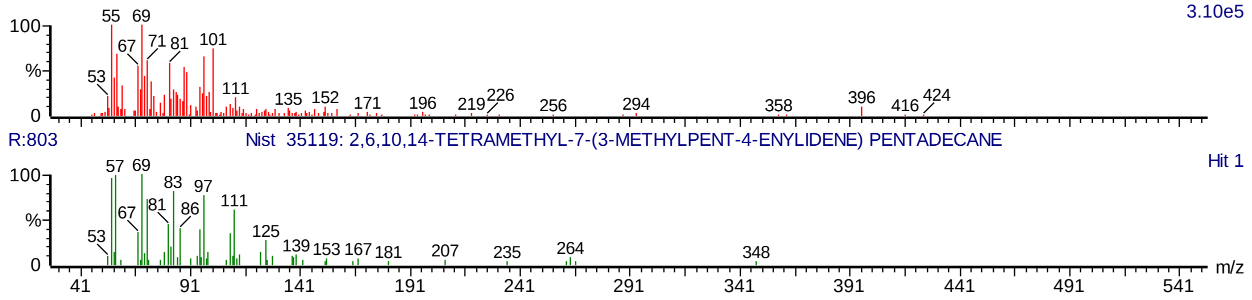

Hit	Rev	For	Compound Name	M.W.	Formula	CAS	Library
1	803	533	2,6,10,14-TETRAMETHYL-7-(3-METHYLPENT-4-	348	$C_{25}H_{48}$	900370-41-6	Nist

80(0.5) 20/m-125(0.5) 3/m-260 (2)Inj255 aux270 , **15-Jun-2021 + 14:55:55**

zahirm800 2250 (17.755)

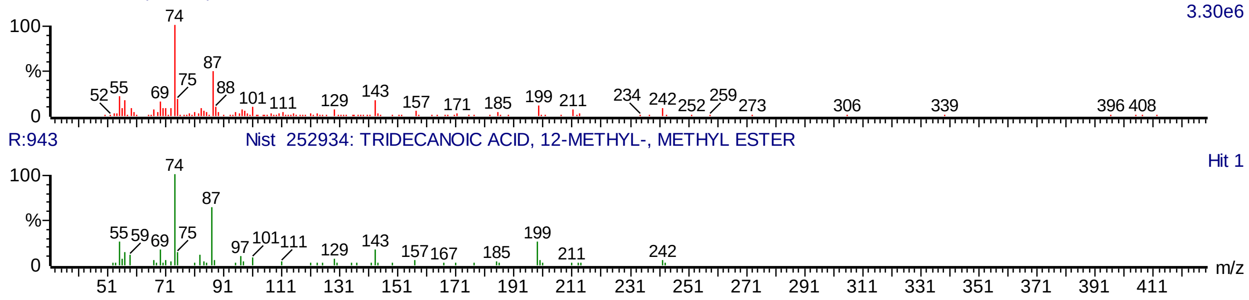

Hit	Rev	For	Compound Name	M.W.	Formula	CAS	Library
1	943	882	TRIDECANOIC ACID, 12-METHYL-, METHYL EST	242	C15H30O2	5129-58-8	Nist

80(0.5) 20/m-125(0.5) 3/m-260 (2)Inj255 aux270 , **15-Jun-2021 + 14:55:55**

zahirm800 2752 (20.266)

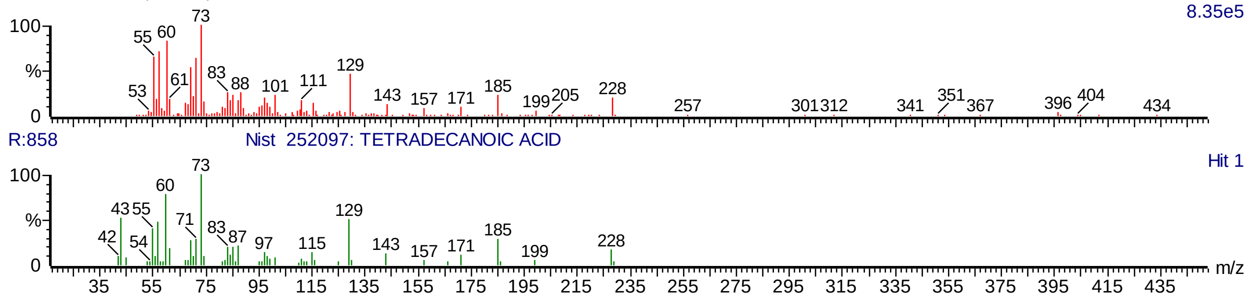

Hit	Rev	For	Compound Name	M.W.	Formula	CAS	Library
1	858	774	TETRADECANOIC ACID	228	C14H28O2	544-63-8	Nist

80(0.5) 20/m-125(0.5) 3/m-260 (2)Inj255 aux270 , **15-Jun-2021 + 14:55:55**

zahirm800 3446 (23.737)

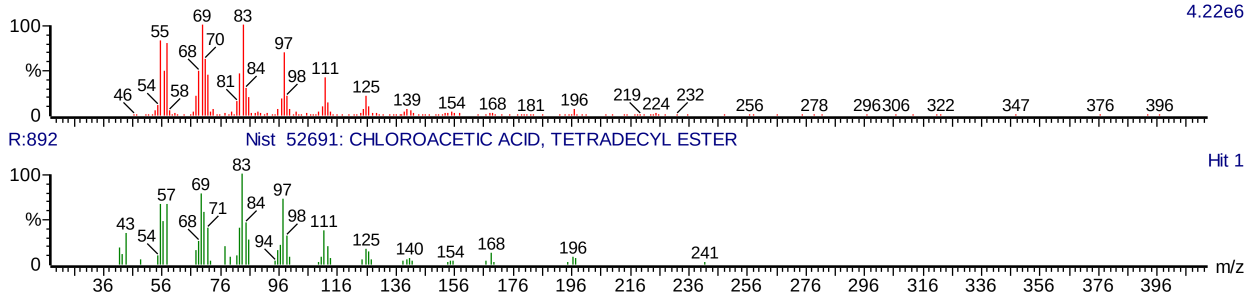

Hit	Rev	For	Compound Name	M.W.	Formula	CAS	Library
1	892 Nist	866	CHLOROACETIC ACID, TETRADECYL ESTER	290	C16H31O2Cl	18277-86-6	

80(0.5) 20/m-125(0.5) 3/m-260 (2)Inj255 aux270 , **15-Jun-2021 + 14:55:55**

zahirm800 3557 (24.292)

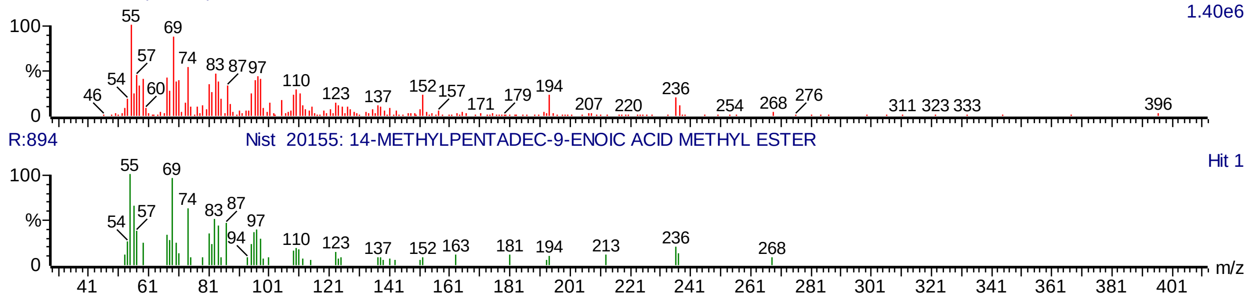

Hit	Rev	For	Compound Name	M.W.	Formula	CAS	Library
1	894	812	14-METHYLPENTADEC-9-ENOIC ACID METHYL ES	268	C17H32O2	900365-89-7	Nist

80(0.5) 20/m-125(0.5) 3/m-260 (2)Inj255 aux270 , **15-Jun-2021 + 14:55:55**

zahirm800 3770 (25.358)

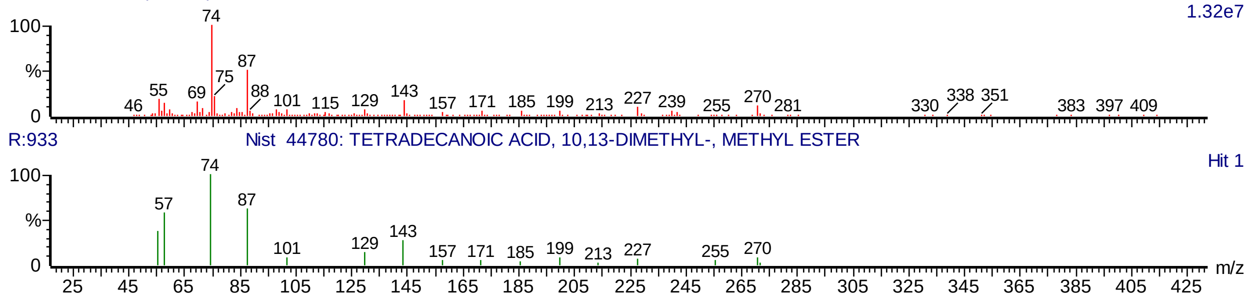

Hit	Rev	For	Compound Name	M.W.	Formula	CAS	Library
1	933	661	TETRADECANOIC ACID, 10,13-DIMETHYL-, MET	270	C17H34O2	267650-23-7	Nist

80(0.5) 20/m-125(0.5) 3/m-260 (2)Inj255 aux270 , **15-Jun-2021 + 14:55:55**

zahirm800 4377 (28.394)

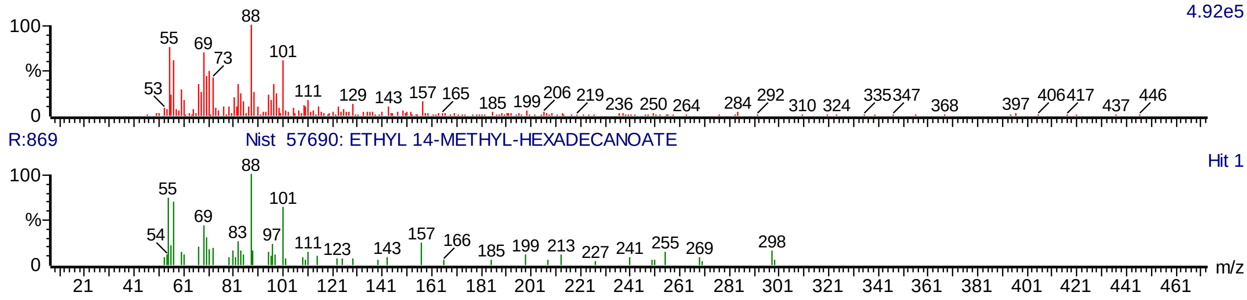

Hit	Rev	For	Compound Name	M.W.	Formula	CAS	Library
1	869	754	ETHYL 14-METHYL-HEXADECANOATE	298	C19H38O2	900336-64-7	Nist

80(0.5) 20/m-125(0.5) 3/m-260 (2)Inj255 aux270 , **15-Jun-2021 + 14:55:55**

zahirm800 5254 (32.781)

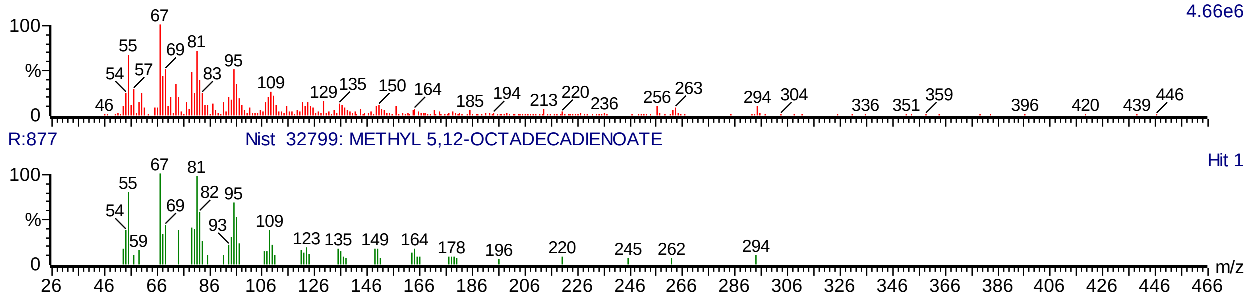

Hit	Rev	For	Compound Name	M.W.	Formula	CAS	Library	
1	877	735	METHYL 5,12-OCTADECADIENOATE			294	C19H34O2	900336-43-1
	Nist							

80(0.5) 20/m-125(0.5) 3/m-260 (2)Inj255 aux270 , **15-Jun-2021 + 14:55:55**

zahirm800 5377 (33.396)

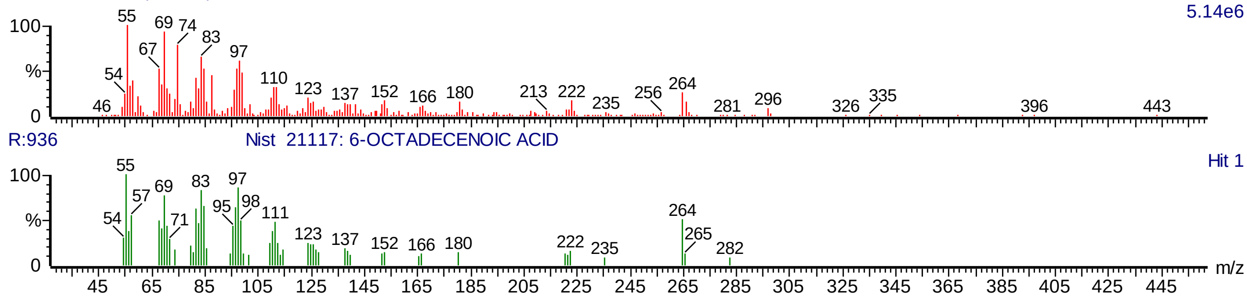

Hit	Rev	For	Compound Name	M.W.	Formula	CAS	Library		
1	936	815	6-OCTADECENOIC ACID		282	C18H34O2		900336-66-8	Nist

80(0.5) 20/m-125(0.5) 3/m-260 (2)Inj255 aux270 , **15-Jun-2021 + 14:55:55**

zahirm800 5686 (34.942)

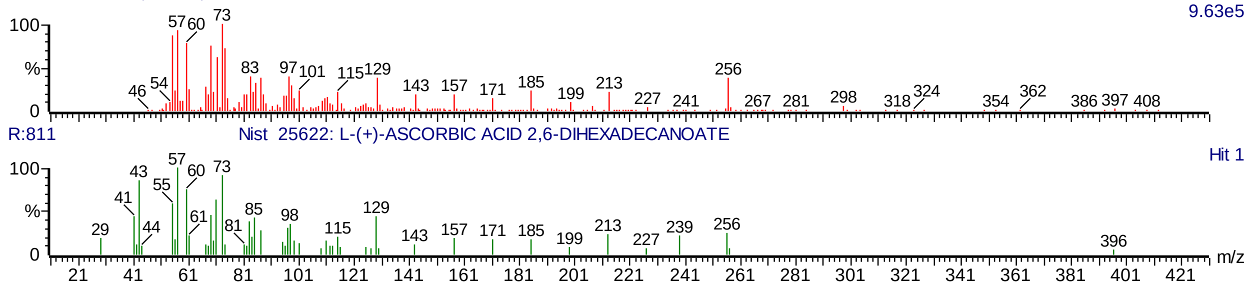

Hit	Rev	For	Compound Name	M.W.	Formula	CAS	Library	
1	811	683	L-(+)-ASCORBIC ACID 2,6-DIHEXADECANOATE			652	C38H68O8	28474-90-0
	Nist							

Figure S1. Chromatograms of M. Indica Peels and Kernel extract

Wrinkles

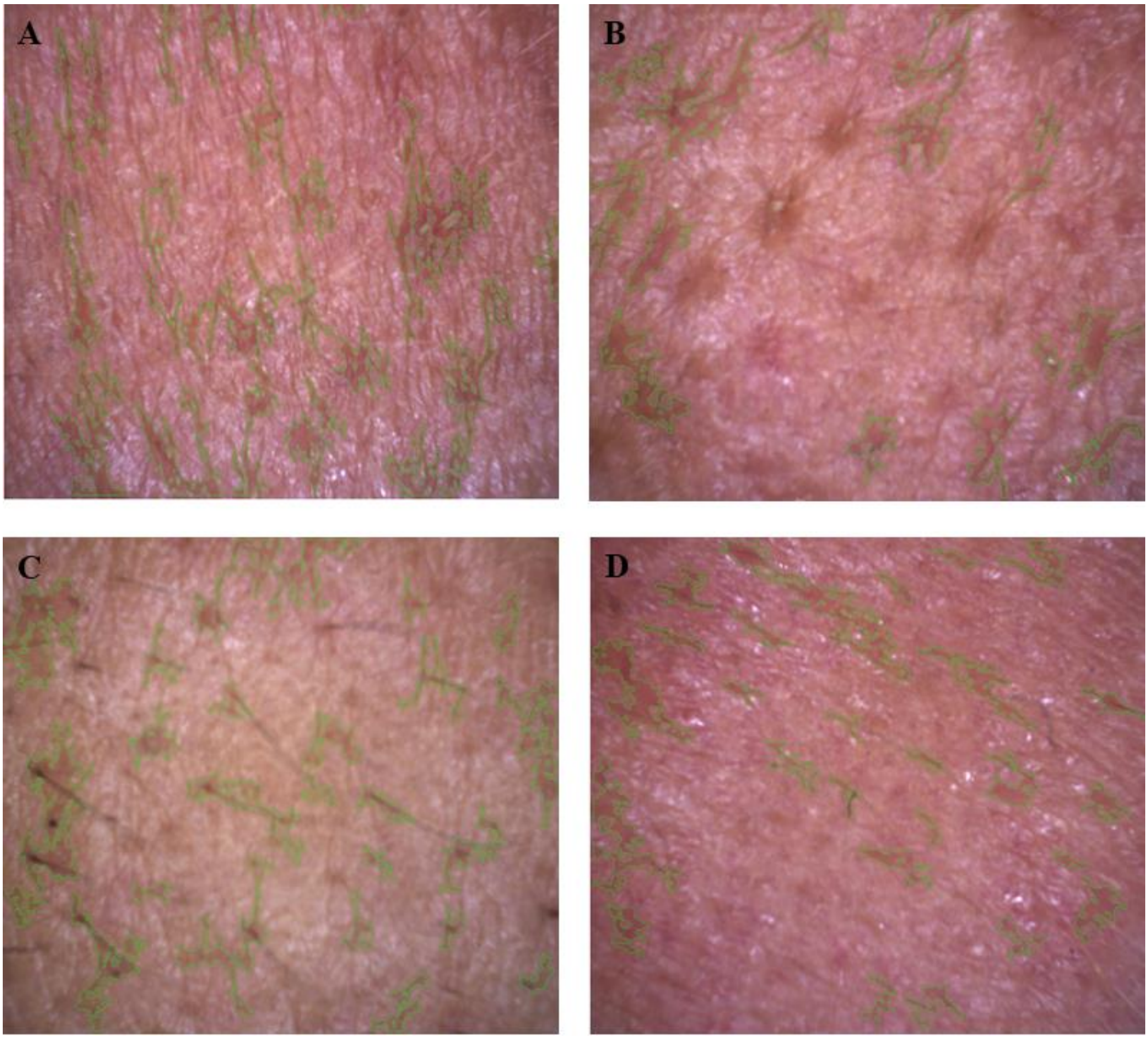

Figure S2. Wrinkle on cheeks of 4 different volunteers. Image was taken by Visio scope attached.

Pores size

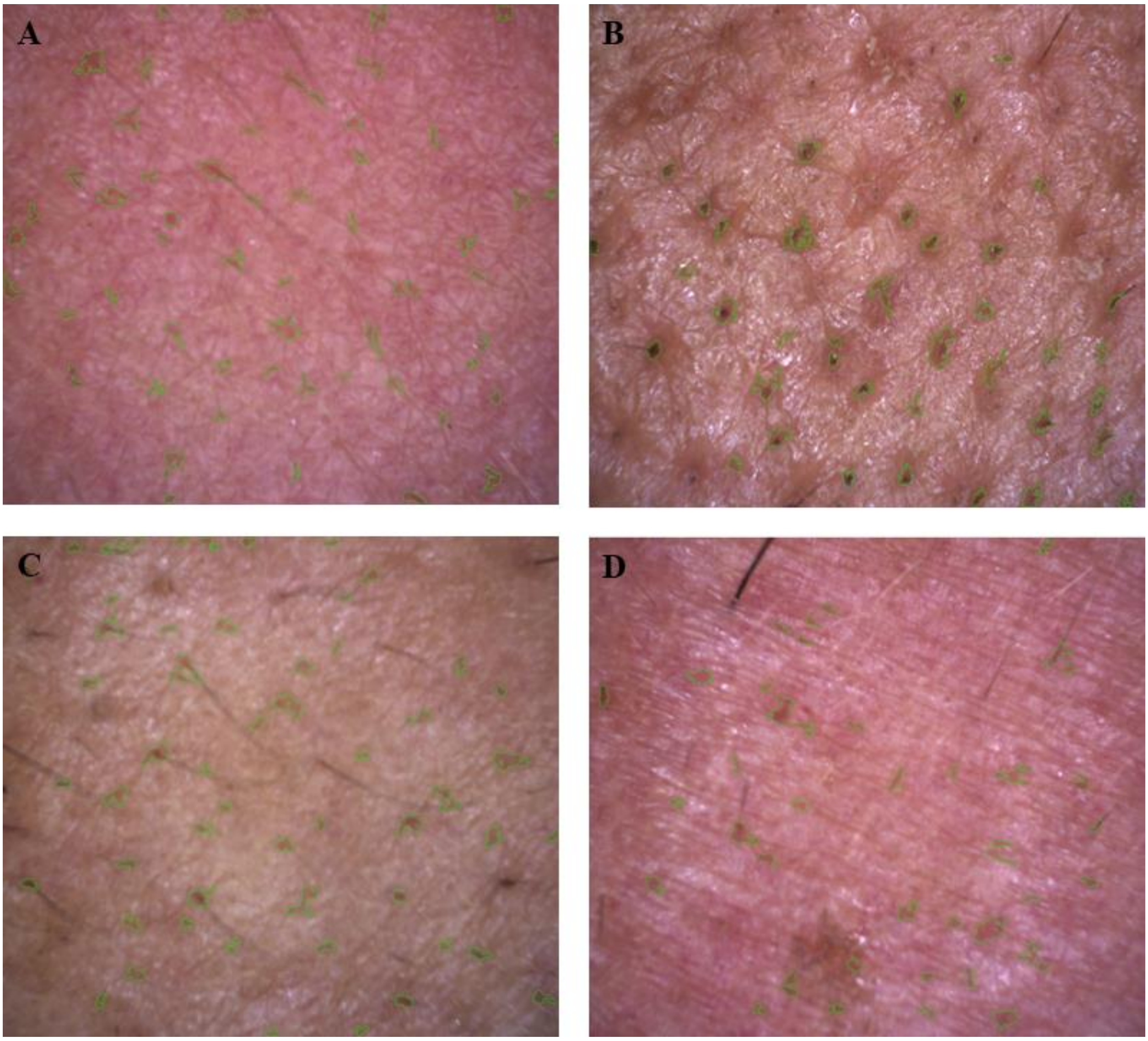

Figure S3. Pores size on of 4 different volunteers. Image was taken by Visio scope attached.

Table S1. Statistical analysis of formulations pH

Dunnett's Multiple Comparisons Test	Mean Diff.	95.00% CI of diff.	Significant?	Summary	Adjusted *p* Value
					**8°C**
1M vs. 2M	-0.2100	-0.5408 to 0.1208	No	ns	0.2521
1M vs. 3M	-0.1600	-0.4908 to 0.1708	No	ns	0.4288
					**25°C**
1M vs. 2M	-0.3100	-0.6408 to 0.02079	No	ns	0.0680
1M vs. 3M	-0.1600	-0.4908 to 0.1708	No	ns	0.4288
					**40°C**
1M vs. 2M	-0.2400	-0.5708 to 0.09079	No	ns	0.1756
1M vs. 3M	-0.1500	-0.4808 to 0.1808	No	ns	0.4712
					**40°C + 75% RH**
1M vs. 2M	0.2200	-0.1108 to 0.5508	No	ns	0.2242
1M vs. 3M	0.1600	-0.1708 to 0.4908	No	ns	0.4288

Dunnett's multiple comparisons test	Mean Diff.	95.00% CI of diff.	Significant?	Summary	Adjusted P Value
**1M**					
8°C vs. 25°C	0.3100	-0.04294 to 0.6629	No	ns	0.0937
8°C vs. 40°C	-0.05000	-0.4029 to 0.3029	No	ns	0.9702
8°C vs. 40°C + 75% RH	-0.5000	-0.8529 to -0.1471	Yes	*	0.0045
**2M**					
8°C vs. 25°C	0.2100	-0.1429 to 0.5629	No	ns	0.3307
8°C vs. 40°C	-0.08000	-0.4329 to 0.2729	No	ns	0.8947
8°C vs. 40°C + 75% RH	-0.07000	-0.4229 to 0.2829	No	ns	0.9253
**3M**					
8°C vs. 25°C	0.3100	-0.04294 to 0.6629	No	ns	0.0937
8°C vs. 40°C	-0.04000	-0.3929 to 0.3129	No	ns	0.9841
8°C vs. 40°C + 75% RH	-0.1800	-0.5329 to 0.1729	No	ns	0.4498

Table S2. Cytotoxicity study on HaCaT cells

Bonferroni's multiple comparisons test	Mean Diff.	95.00% CI of diff.	Significant?	Summary	Adjusted P Value
**M-Ext - M-NLC**					
0	0.000	-6.258 to 6.258	No	ns	>0.9999
1	-0.5000	-6.758 to 5.758	No	ns	>0.9999
10	-7.100	-13.36 to -0.8419	Yes	*	0.0188
20	-6.400	-12.66 to -0.1419	Yes	*	0.0425
50	-7.700	-13.96 to -1.442	Yes	**	0.0092
100	-10.60	-16.86 to -4.342	Yes	***	0.0002
250	-9.900	-16.16 to -3.642	Yes	***	0.0006

Table S3. Cytotoxicity study on Fibroblast cells

Bonferroni's multiple comparisons test	Mean Diff.	95.00% CI of diff.	Significant?	Summary	Adjusted P Value
**M-Ext - M-NLC**					
0	0.000	-5.904 to 5.904	No	ns	>0.9999
1	-2.100	-8.004 to 3.804	No	ns	>0.9999
10	-3.000	-8.904 to 2.904	No	ns	>0.9999
20	-7.100	-13.00 to -1.196	Yes	*	0.0113
50	-9.700	-15.60 to -3.796	Yes	***	0.0004
100	-9.000	-14.90 to -3.096	Yes	***	0.0009
250	-9.700	-15.60 to -3.796	Yes	***	0.0004

Table S4. Statistical analysis of permeation studies

Bonferroni's multiple comparisons test	Mean Diff.	95.00% CI of diff.	Significant?	Summary	Adjusted P Value
**M-Ext - M-NLC**					
1	-0.1000	-0.1378 to -0.06220	Yes	****	<0.0001
2	-0.1100	-0.1478 to -0.07220	Yes	****	<0.0001
3	-0.1500	-0.1878 to -0.1122	Yes	****	<0.0001
6	-0.1800	-0.2178 to -0.1422	Yes	****	<0.0001

Table S5. Statistical analysis of Erythema

Dunnett's multiple comparisons test	Mean Diff.	95.00% CI of diff.	Significant?	Summary	Adjusted P Value
B-NLC-E					
M1 vs. M2	0.8600	0.5186 to 1.201	Yes	****	<0.0001
M1 vs. M3	0.7300	0.3886 to 1.071	Yes	****	<0.0001
M-NLC-E					
M1 vs. M2	5.300	4.959 to 5.641	Yes	****	<0.0001
M1 vs. M3	12.55	12.21 to 12.89	Yes	****	<0.0001

Table S6. Statistical analysis of Skin melanin level

Dunnett's multiple comparisons test	Mean Diff.	95.00% CI of diff.	Significant?	Summary	Adjusted P Value
B-NLC-E					
1M vs. 2M	-0.2300	-0.3282 to -0.1318	Yes	****	<0.0001
1M vs. 3M	-0.5600	-0.6582 to -0.4618	Yes	****	<0.0001
M-NLC-E					
1M vs. 2M	1.900	1.802 to 1.998	Yes	****	<0.0001
1M vs. 3M	11.64	11.54 to 11.74	Yes	****	<0.0001

Table S7. Statistical analysis of TEWL index

Dunnett's multiple comparisons test	Mean Diff.	95.00% CI of diff.	Significant?	Summary	Adjusted P Value
B-NLC-E					
1M vs. 2M	0.3800	0.1827 to 0.5773	Yes	***	0.0001
1M vs. 3M	0.5300	0.3327 to 0.7273	Yes	****	<0.0001
M-NLC-E					
1M vs. 2M	1.210	1.013 to 1.407	Yes	****	<0.0001
1M vs. 3M	12.92	12.72 to 13.12	Yes	****	<0.0001

Table S8. Statistical analysis of Moisture level

Dunnett's multiple comparisons test	Mean Diff.	95.00% CI of diff.	Significant?	Summary	Adjusted P Value
B-NLC-E					
1M vs. 2M	-0.6200	-0.8343 to -0.4057	Yes	****	<0.0001
1M vs. 3M	-1.730	-1.944 to -1.516	Yes	****	<0.0001
M-NLC-E					
1M vs. 2M	-1.290	-1.504 to -1.076	Yes	****	<0.0001
1M vs. 3M	-4.650	-4.864 to -4.436	Yes	****	<0.0001

Table S9. Statistical analysis of Sebum level

Dunnett's multiple comparisons test	Mean Diff.	95.00% CI of diff.	Significant?	Summary	Adjusted P Value
B-NLC-E					
M1 vs. M2	2.460	2.328 to 2.592	Yes	****	<0.0001
M1 vs. M3	0.7900	0.6579 to 0.9221	Yes	****	<0.0001
M-NLC-E					
M1 vs. M2	3.660	3.528 to 3.792	Yes	****	<0.0001
M1 vs. M3	18.29	18.16 to 18.42	Yes	****	<0.0001

Table S10. Statistical analysis of elasticity of skin

Dunnett's multiple comparisons test	Mean Diff.	95.00% CI of diff.	Significant?	Summary	Adjusted P Value
B-NLC-E					
1M vs. 2M	1.720	1.531 to 1.909	Yes	****	<0.0001
1M vs. 3M	0.2300	0.04137 to 0.4186	Yes	*	0.0144
M-NLC-E					
1M vs. 2M	-3.150	-3.339 to -2.961	Yes	****	<0.0001
1M vs. 3M	-13.12	-13.31 to -12.93	Yes	****	<0.0001

Table S11. Statistical analysis of Wrinkles

Dunnett's multiple comparisons test	Mean Diff.	95.00% CI of diff.	Significant?	Summary	Adjusted P Value
B-NLC-E					
1M vs. 2M	-3.600	-3.756 to -3.444	Yes	****	<0.0001
1M vs. 3M	-9.650	-9.806 to -9.494	Yes	****	<0.0001
M-NLC-E					
1M vs. 2M	0.5900	0.4343 to 0.7457	Yes	****	<0.0001
1M vs. 3M	10.61	10.45 to 10.77	Yes	****	<0.0001

Table S12. Statistical analysis of pH of the skin

Dunnett's multiple comparisons test	Mean Diff.	95.00% CI of diff.	Significant?	Summary	Adjusted P Value
B-NLC-E					
1M vs. 2M	0.1200	0.08500 to 0.1550	Yes	****	<0.0001
1M vs. 3M	0.2500	0.2150 to 0.2850	Yes	****	<0.0001
M-NLC-E					
1M vs. 2M	0.000	-0.03500 to 0.03500	No	ns	>0.9999
1M vs. 3M	0.5400	0.5050 to 0.5750	Yes	****	<0.0001

Table S13. Statistical analysis of Pores sizes

Dunnett's multiple comparisons test	Mean Diff.	95.00% CI of diff.	Significant?	Summary	Adjusted P Value
B-NLC-E					
1M vs. 2M	-3.600	-3.897 to -3.303	Yes	****	<0.0001
1M vs. 3M	-9.650	-9.947 to -9.353	Yes	****	<0.0001
M-NLC-E					
1M vs. 2M	0.5900	0.2927 to 0.8873	Yes	****	<0.0001
1M vs. 3M	10.61	10.31 to 10.91	Yes	****	<0.0001

Table S14. Comparison of measuring options for B-NLC-E and M-NLC-E

**Measuring options**	**Mean values**				
	**Formulation**	**0 hr**	**1 M**	**2 M**	**3 M**
**Skin melanin**	B-NLC-E	24.33	24.30	24.38	24.09
	M-NLC-E	24.39	21.61	21.02	18.73
**Skin erythema**	B-NLC-E	52.69	52.35	51.76	51.92
	M-NLC-E	56.82	50.57	47.25	43.00
**Moisture**	B-NLC-E	41.48	41.41	41.29	41.58
	M-NLC-E	45.82	53.64	53.95	55.95
**TEWL**	B-NLC-E	14.09	14.09	14.00	14.00
	M-NLC-E	14.09	12.64	12.45	10.82
**Skin pH**	B-NLC-E	4.79	4.78	4.77	4.76
	M-NLC-E	4.75	4.78	4.78	4.75
**Skin Elasticity**	B-NLC-E	67.64	67.73	66.36	67.45
	M-NLC-E	67.09	69.27	71.45	78.09
**Skin Sebum**	B-NLC-E	15.91	15.36	15.00	15.18
	M-NLC-E	12.63	10.63	10.18	8.36

Written consent sample

COMSATS UNIVERSITY ISLAMABAD, ABBOTTABAD CAMPUS
CONSENT TO PARTICIPATE IN RESEARCH STUDY
Short Form Written Consent (used with oral consent form)

STUDY TITLE: "Complete Skin Investigation of *Mangifera indica* L. Extract Loaded Nano-lipid Carriers Green Topical Formulation" on skin of volunteers in Population of Abbottabad.

Principle Investigator: Dr. Atif Ali

Investigator: Zaheer Ullah Khan

WRTTIEN CONCENT

I confirm that the researcher has explained the elements of informed consent to the participant. The subject knows that their participation is voluntary, and that they do not need to answer all questions. The purpose of the research as well as the risks and benefits have been explained. The procedures as well as the time commitment have been outlined. The participant understands issues of confidentiality.

Participant name ________________________

Participant signature__________________________
